# Prominent Indomethacin-Induced Enteropathy in Fcgriib *Defi-cient lupus* Mice: An Impact of Macrophage Responses and Immune Deposition in Gut

**DOI:** 10.3390/ijms22031377

**Published:** 2021-01-29

**Authors:** Thansita Bhunyakarnjanarat, Kanyarat Udompornpitak, Wilasinee Saisorn, Bhumdhanin Chantraprapawat, Peerapat Visitchanakun, Cong Phi Dang, Jiraphorn Issara-Amphorn, Asada Leelahavanichkul

**Affiliations:** 1 Medical Microbiology, Interdisciplinary and International Program, Graduate School, Chulalongkorn University, Bangkok 10330, Thailand; thansitadew@gmail.com; 2 Department of Microbiology, Faculty of Medicine, Chulalongkorn University, Bangkok 10330, Thailand; 3 Translational Research in Inflammation and Immunology Research Unit (TRIRU), Department of Microbiology, Chulalongkorn University, Bangkok 10330, Thailand; jubjiibb@hotmail.com (K.U.); wsaisorn@gmail.com (W.S.); martfreeee@docchula.com (B.C.); peerapat.visitchanakun@gmail.com (P.V.); pilotdang1308@gmail.com (C.P.D.); jiraphorn298@gmail.com (J.I.-A.); 4 Department of Microbiology, Immunology Unit, Chulalongkorn University, Bangkok 10330, Thailand

**Keywords:** FcgRIIb deficient mice, systemic lupus erythematosus, NSAIDs-enteropathy, gut leakage

## Abstract

A high dose of NSAIDs, a common analgesic, might induce lupus activity through several NSAIDs adverse effects including gastrointestinal permeability defect (gut leakage) and endotoxemia. Indomethacin (25 mg/day) was orally administered for 7 days in 24-wk-old Fc gamma receptor IIb deficient (FcgRIIb-/-) mice, an asymptomatic lupus model (increased anti-dsDNA without lupus nephritis), and age-matched wild-type (WT) mice. Severity of indomethacin-induced enteropathy in FcgRIIb-/- mice was higher than WT mice as demonstrated by survival analysis, intestinal injury (histology, immune-deposition, and intestinal cytokines), gut leakage (FITC-dextran assay and endotoxemia), serum cytokines, and lupus characteristics (anti-dsDNA, renal injury, and proteinuria). Prominent responses of FcgRIIb-/- macrophages toward lipopolysaccharide (LPS) compared to WT cells due to the expression of only activating-FcgRs without inhibitory-*FcgRIIb* were demonstrated. Extracellular flux analysis indicated the greater mitochondria activity (increased respiratory capacity and respiratory reserve) in FcgRIIb-/- macrophages with a concordant decrease in glycolysis activity when compared to WT cells. In conclusion, gut leakage-induced endotoxemia is more severe in indomethacin-administered FcgRIIb-/- mice than WT, possibly due to the enhanced indomethacin toxicity from lupus-induced intestinal immune-deposition. Due to a lack of inhibitory-*FcgRIIb* expression, mitochondrial function, and cytokine production of FcgRIIb-/- macrophages were more prominent than WT cells. Hence, lupus disease-activation from NSAIDs-enteropathy-induced gut leakage is possible.

## 1. Introduction

Systemic lupus erythematosus (SLE) is a common autoimmune disease caused by a complex mixture of genetic and environmental factors and Fc gamma receptor IIb deficient (FcgRIIb-/-) mice have been used as a representative lupus model. In Asian populations, the prevalence of a dysfunctional polymorphism of FcgRIIb, the only inhibitory receptor among the FcgR family [1,2,3], is high [4] and FcgRIIb-/- mice have been used as a lupus mouse model [5,6,7]. There is an age-dependency in the development of lupus characteristics in FcgRIIb-/- mice, as anti-dsDNA, a major lupus auto-antibody, spontaneously develops in these mice as early as 16–24 wks old [5,6,7]. FcgRIIb-/- mice younger than 24 wks old are asymptomatic because of the undetectable anti-dsDNA [8,9]. The loss of inhibitory signaling in FcgRIIb-/- mice not only causes lupus, but also results in the hyperresponsiveness against pathogen molecules, including lipopolysaccharide (LPS). This is possibly due to the crosstalk of FcgRs (a receptor for Fc portion of immunoglobulin) and TLR-4 (a LPS receptor) [5,8,9,10,11].

In addition to LPS from Gram-negative bacterial cell wall, TLR-4 also recognizes other pathogen associated molecular patterns (PAMPs) from other organisms and damage-associated molecular patterns (DAMPs) from of the damaged host cells [12,13]. Hence, the loss of inhibitory FcgRIIb might enhance the reaction against either molecules from pathogens or host cells due to the FcgRs-TLR-4 crosstalk [14]. Indeed, endotoxin (LPS), from mucosal immune complex deposition induced gut permeability defect [15], prominently affects FcgRIIb-/- mice compared to wild-type (WT) despite nonprominent gastrointestinal (GI) symptoms [5,16]. Without the inhibitory FcgRIIb, TLR-4 cross-links only to the activating FcgRs [5,17,18]. In addition, macrophages, major immune cells, recognize LPS through TLR-4 [19,20,21] and FcgRs [1,2,3], the innate- and adaptive-immune receptors, respectively. Hence, the physiologic alteration of the receptors between WT and FcgRIIb-/- cells may differ.

Nonsteroidal anti-inflammatory drugs (NSAIDs) are commonly used to relieve several symptoms (musculoskeletal pain and arthritis) in patients with active autoimmune diseases. The anti-inflammatory property of NSAIDs bases on the blockage of cyclooxygenase (COX), also referred to as prostaglandin-endoperoxide synthase, to prevent prostaglandins (PGs) conversion from arachidonic acid, a cell membrane polyunsaturated phospholipid [22]. As such, COX-1 is a housekeeping enzyme for maintaining several functions including blood flow in several organs (kidney and gut), lung function (airway smooth muscle), intestinal mucosa, and platelet functions [22]. Meanwhile, COX-2 is an enzyme for the synthesis of several proinflammatory-PGs including in macrophages/monocytes [23,24]. Hence, the COX blockage results in several NSAIDs adverse effects, mainly through the smooth muscle contraction (vasospasm and bronchospasm) and the mucosal injury [25,26]. As the inflammatory reaction is an important part of the wound healing process [27] that could be severe enough for the induction of systemic inflammation (partly through endotoxemia) [28], and the enhanced inflammatory responses (cytokines, endotoxemia, and cell apoptosis) due to the NSAIDs adverse effects are mentioned [22], the increased inflammatory activity from NSAIDs is possible.

Among all NSAIDs side effects, intestinal injury and nephropathy are the most common complications [29]. Although gastritis is the most common NSAIDs-induced enteropathy, NSAIDs also damage gut mucosal throughout the GI tract [29] and cause what is referred to as gut leakage or leaky gut syndrome [29,30]. NSAID-induced enteropathy, including gut leakage, is a well-known negative side effect [29,30]. Additionally, NSAIDs cause nephropathy [31,32] and renal injury induces systemic inflammation through the gut–renal axis, partly from endotoxemia [33]. Since inflammation and gut leakage induce lupus flare-up and lupus activity [34,35,36], it is possible that NSAIDs might activate lupus disease activity through NSAIDs-induced gut leakage. Despite an availability of the selective COX-2 inhibitory NSAIDs with a lower GI side effect, the short-acting nonselective COX-1 and COX-2 inhibitory NSAIDs are still currently used in several situations [37,38,39]. Indomethacin, a potent NSAID with a high likelihood of a GI side effect, continues to be administered to patients [40,41] and is frequently employed in animal models [42,43]. Here, our study performed both in vitro and in vivo investigations to determine the impact of indomethacin, a representative NSAID, against lupus using FcgRIIb-/- asymptomatic lupus mice at 24 wks old.

## 2. Results

A high dose of indomethacin produced more severe intestinal ulcers and enhanced gastro-intestinal permeability defect (gut leakage) in 24-wk-old FcgRIIb-/- mice compared to wild-type (WT) mice suggesting a prominent adverse effect of NSAIDs in lupus.

### 2.1. Prominent Indomethacin-Induced Renal Injury, Enterocolitis, and Endotoxemia in FcgRIIb-/-Mice Compared with Wild-Type Mice

**Figure 1:**
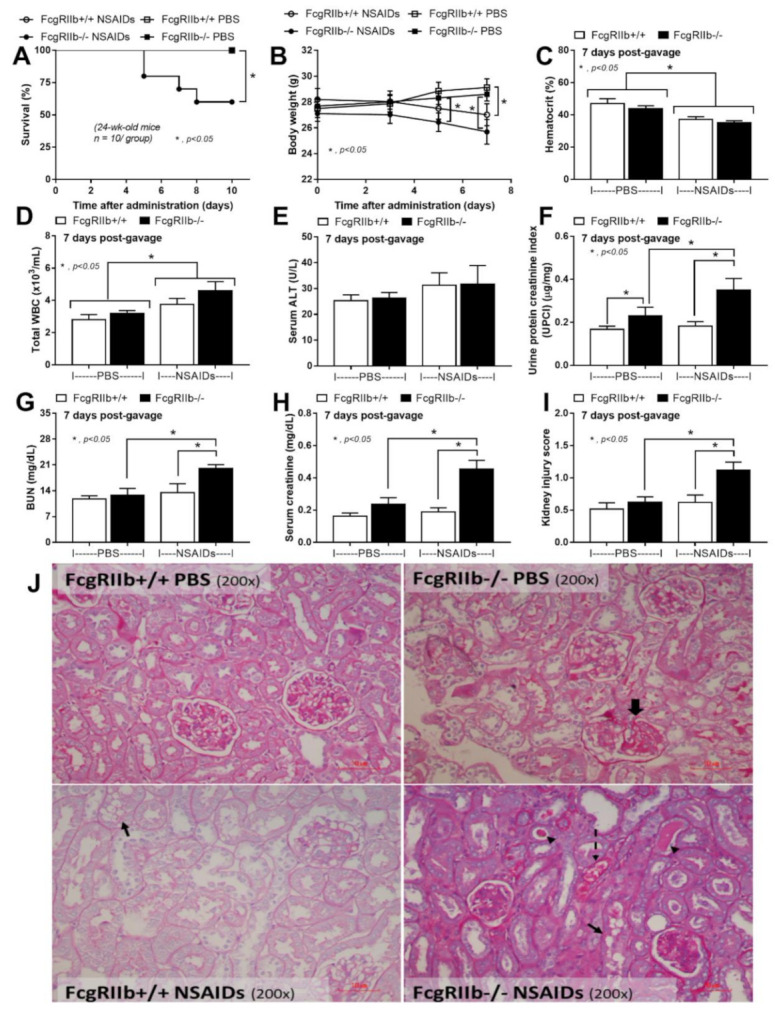
Characteristics of mice after the administration of indomethacin (NSAIDs) or phosphate buffer solution (PBS) control in FcgRIIb-/- lupus mice and wild-type (FcgRIIb+/+) mice as indicated by survival analysis (**A**), body weight alteration (**B**), and the organ injury at 7 days of the administration as indicated by hematocrit (**C**), total white blood cell (WBC) count in blood (**D**), liver injury by serum alanine transaminase (**E**), and renal injury using urine protein creatinine index (UPCI), blood urea nitrogen (BUN), serum creatinine, and renal injury score with the representative Periodic acid-Schiff staining (PAS) histological pictures (original magnification 200×) (**F**–**J**) are demonstrated (*n* = 7–10/time point or group). Thick arrow, prominent mesangial staining in FcgRIIb-/- PBS; thin arrows, NSAIDs-induced tubular vacuolization in both mouse strains; arrow heads, proteinaceous cast formation in renal tubule of NSAIDs administered FcgRIIb-/- mice; dotted-line arrow, red blood cell casts in renal tubule of NSAIDs administered FcgRIIb-/- mice.

**Figure 2:**
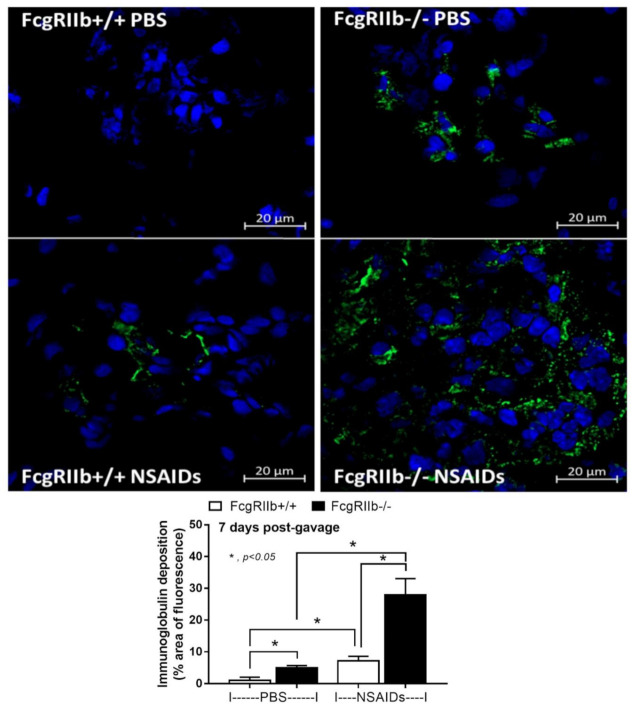
Representative immunohistochemistry staining (original magnification 200×) of immunoglobulin (green color) and cell nuclei using 4′,6-diamidino-2-phenylindole (DAPI) (blue color) in glomeruli from mice with the administration of indomethacin (NSAIDs) or phosphate buffer solution (PBS) control in FcgRIIb-/- lupus mice and wild-type (FcgRIIb+/+) mice at 7 days of the administration with the immunofluorescent score (*n* = 5–7/group) are demonstrated.

Indomethacin at 25 mg/kg/day produced a 40% mortality rate in FcgRIIb-/- mice, versus zero mortality in WT mice without a difference in the body weight between mouse strains (Figure 1A,B). Although all mice with NSAIDs demonstrated positive occult blood test (data not shown) with the reduced bodyweight in both mouse strains (Figure 1B), there was no hematochezia and no change in fecal color (data not shown). However, NSAIDs similarly reduced hematocrit and increased total white blood cell count in both mouse strains without the liver injury (Figure 1C–E). Renal injury at 7 days post-NSAIDs was more prominent in FcgRIIb-/- mice compared with the WT as indicated by proteinuria, blood urea nitrogen, serum creatinine, renal histological score, and glomerular immune complex (IC) deposition ( 1F–J and 2). Interestingly, the common findings in lupus nephritis [36] including proteinaceous casts, red blood cell casts (Figure 1J; arrow heads and dotted line arrow) and glomerular IC deposition (Figure 2) at 7 days of the experiments were prominently presented in FcgRIIb-/- mice, but neither WT mice nor PBS-control FcgRIIb-/- mice, suggesting an exacerbation of lupus activity by NSAIDs. Notably, slightly elevated proteinuria (Figure 1F) and prominent mesangial staining (Figure 1J, thick arrow) was demonstrated in PBS-control FcgRIIb-/- mice without renal injury by other parameters (Figure 1G–J), implying a pre-existing lupus-induced renal injury in 24-wk-old FcgRIIb-/- mice.

**Figure 3:**
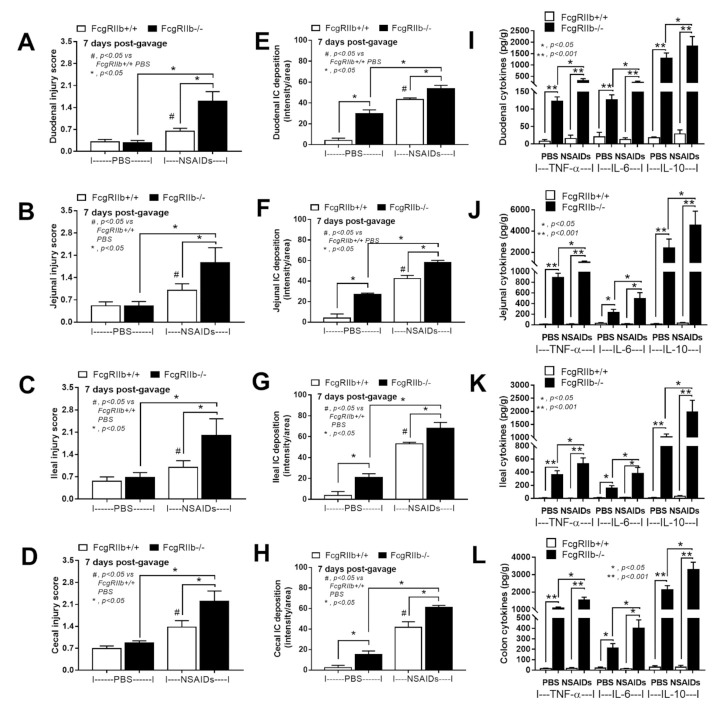
Characteristics of intestinal injury in FcgRIIb-/- lupus mice and wild-type (FcgRIIb+/+) mice after the administration of indomethacin (NSAIDs) or phosphate buffer solution (PBS) control as determined by intestinal histopathological scores (**A**–**D**), immune deposition (**E–H**), and intestinal cytokines (**I–L**) (*n* = 6–8/group).

**Figure 4:**
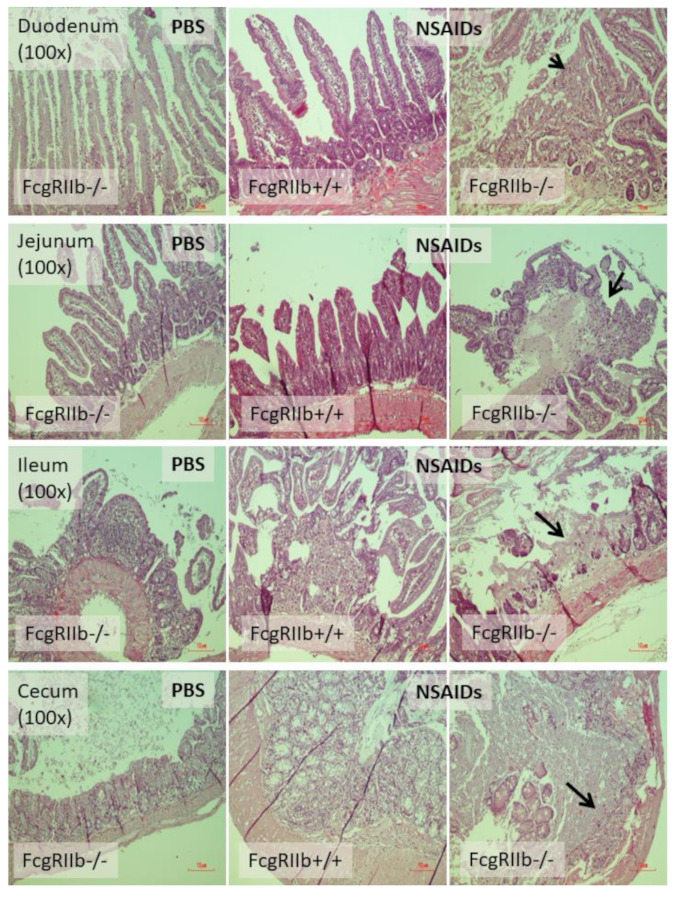
Representative figures of intestinal histology (hematoxylin and eosin staining) of FcgRIIb-/- lupus mice and wild-type (FcgRIIb+/+) mice after the administration of indomethacin (NSAIDs) or phosphate buffer solution (PBS) control (original magnification 200×) are demonstrated. of PBS-administered wild-type control mice (FcgRIIb+/+ PBS) were not demonstrated due to the similarity to FcgRIIb-/- PBS control mice. Arrow, raw surface of the intestinal mucosa indicates the intestinal ulcers.

**Figure 5:**
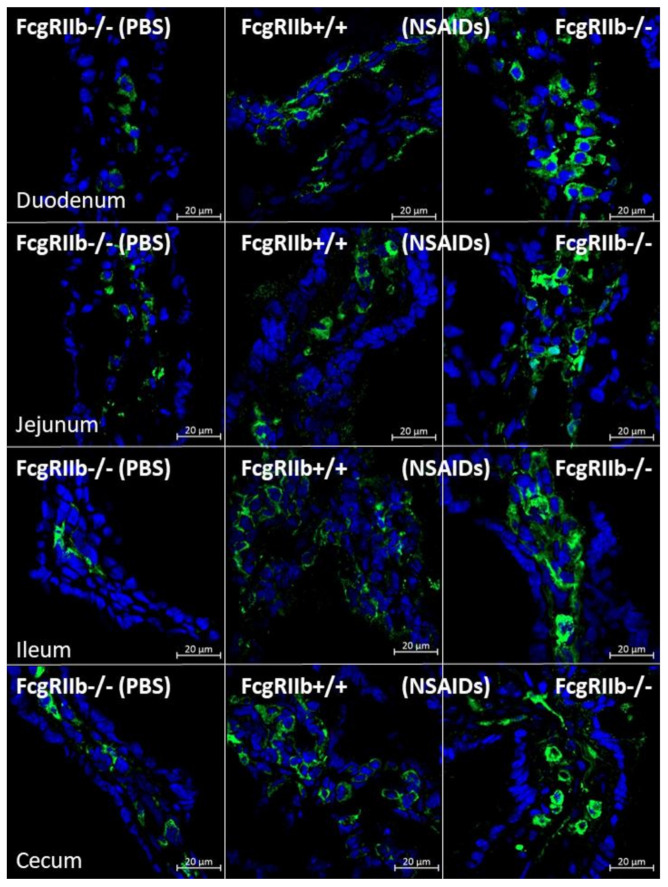
Representative figures of immune deposition in the intestines of FcgRIIb-/- lupus mice and wild-type (FcgRIIb+/+) mice after the administration of indomethacin (NSAIDs) or phosphate buffer solution (PBS) control (original magnification 200×) are demonstrated. of PBS-administered wild-type control mice (FcgRIIb+/+ PBS) were not demonstrated due to the non-detectability of immune deposition. Green and blue colors demonstrate mouse IgG and intestinal nuclei, respectively.

**Figure 6:**
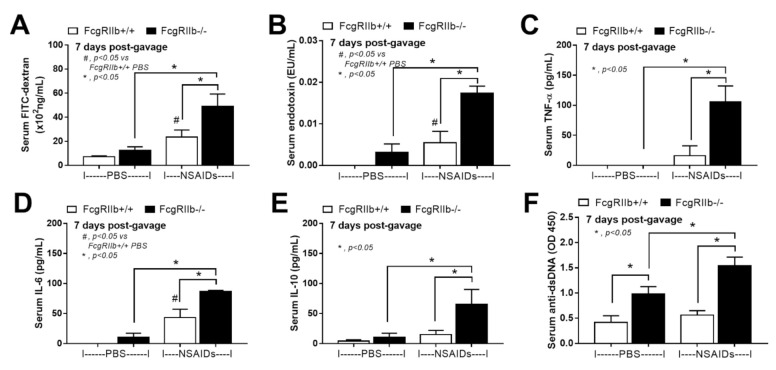
Characteristics of FcgRIIb-/- lupus mice and wild-type (FcgRIIb+/+) mice after the administration of indomethacin (NSAIDs) or phosphate buffer solution (PBS) control as determined by gut leakage (FITC-dextran assay) (**A**), endotoxemia (**B**), serum cytokines (**C–E**), and anti-dsDNA (**F**) (*n* = 5–7/group).

In parallel, the severity of enterocolitis in FcgRIIb-/- mice was more prominent throughout the intestines from duodenum to colon (Figure 3A–D and Figure 4) compared to the WT mice. The ulceration wounds were detectable in the duodenum, jejunum, ileum, and colon of NSAIDs-administered FcgRIIb-/- mice (Figure 4, arrows), while only mononuclear cells infiltration was found in WT mice (Figure 4). In parallel, the immune deposition in the intestine of the control FcgRIIb-/- mice (non-NSAIDs administration) was detectable in FcgRIIb-/- mice, but not in WT (Figure 3E–H and Figure 5). There was no clinical manifestation of GI abnormality as evidenced by similar bodyweight of FcgRIIb-/- vs. WT mice at baseline (0 h time-point) (Figure 1B), despite a mild immune deposition in the gut of lupus mice with PBS control (Figure 3E–H and 5). After NSAIDs administration, immunoglobulin (Ig) was also detectable in the intestines of WT mice indicating the Ig of wound repairing processes [44]. However, the immunoglobulin intensity in NSAIDs-administered WT mice was less than in NSAIDs-administered FcgRIIb-/- mice ( 3E–H and 5), possibly due to the immune deposition before NSAIDs administration in asymptomatic lupus mice. In addition, cytokines from the intestinal tissue of NSAIDs-administered FcgRIIb-/- were higher than NSAIDs-administered WT mice, while the cytokine levels exhibited no difference between mouse strains in the control groups (Figure 3I–L). After NSAIDs administration, gut permeability defect (gut-leakage) as determined by FITC-dextran assay and endotoxemia was higher in FcgRIIb-/- mice than WT mice (Figure 6A,B). Gut leakage was not detectable in FcgRIIb-/- control mice (Figure 6A), despite the detectable immune deposition (Figure 3E–J), supporting asymptomatic immune deposition in the gut of the lupus mice. Unsurprisingly, the endotoxemia-induced systemic inflammation in NSAIDs-administered FcgRIIb-/- mice was more severe than in WT (Figure 6C–E). Administration of 7 days of indomethacin induced systemic inflammation and raised the level of anti-dsDNA in FcgRIIb-/- mice (Figure 6F) that possibly exacerbate lupus nephritis as determined by renal function and cast formation in renal histology (Figure 1F–J). In contrast, NSAIDs did not induce anti-dsDNA in WT mice (Figure 6F).

### 2.2. Prominent Responses against Endotoxin in FcgRIIb-/- Macrophages Compared to Wild-Type Macrophages In Vitro Implied Less Impact of LPS Tolerant Macrophages in NSAIDs-Administered Mice

**Figure 7:**
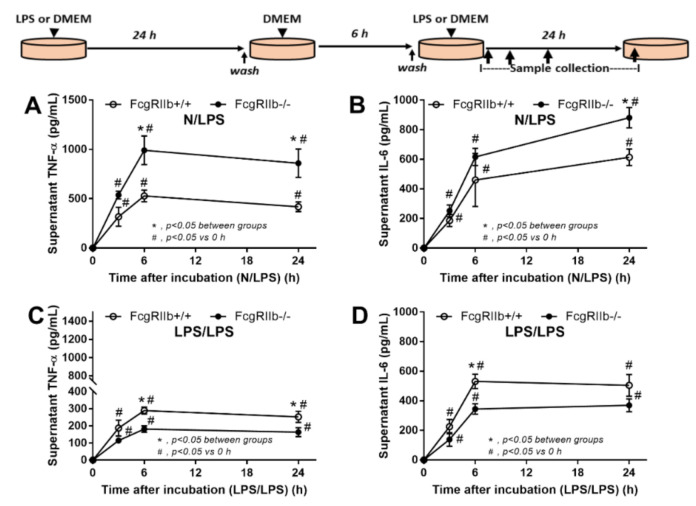
Schema of the in vitro experiments (see method) is demonstrated (upper part of figure). Supernatant cytokines secreted by macrophages from FcgRIIb-/- lupus mice and wild-type (FcgRIIb+/+) mice after 6 h incubation with a single lipopolysaccharide stimulation (N/LPS) (**A**,**B**) or stimulation twice (LPS/LPS) (**C**,**D**) are demonstrated. Independent triplicate experiments were performed.

**Figure 8:**
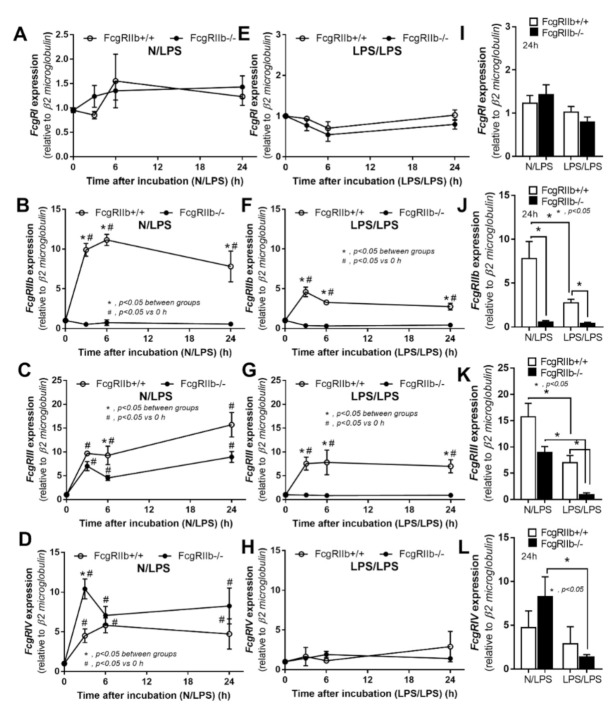
The gene expression by quantitative polymerase chain reaction in macrophages from FcgRIIb-/- lupus mice and wild-type (FcgRIIb+/+) mice after 6 h incubation with a single lipopolysaccharide stimulation (N/LPS) (**A–D**) or stimulation twice (LPS/LPS) (**E–H**) and the presentation in graphs of the 24 h post-stimulation (**I**–**L**) are demonstrated. Independent triplicate experiments were performed.

As LPS from gut translocation might activate macrophages in either acute or chronic exposure manners, the in vitro LPS stimulation was performed with a single (N/LPS) and a repeated activation (LPS/LPS). As such, the hyperinflammatory response of FcgRIIb-/- macrophages was demonstrated by the higher TNF-α and IL-6 in supernatant after a single LPS stimulation (N/LPS) (Figure 7A,B) supporting a previous publication [10]. With the repeated LPS stimulation (LPS/LPS), the levels of supernatant cytokines from FcgRIIb-/- macrophages were lower than in WT cells although higher than the baseline levels (Figure 7C,D). This indicates the potent response against a single LPS stimulation and the prominent endotoxin tolerance in FcgRIIb-/- macrophages compared to WT cells. Since FcgRs consists of both activating and inhibitory receptors [45] and the cross-link between FcgRs and TLR-4, the endotoxin receptor, is mentioned [46], the different response of macrophages between the single vs. repeated LPS stimulation might be due to the different expression of FcgRs. The gene expression of *FcgRIIb*, an inhibitory receptor, was determined along with other activating *FcgRs*. Accordingly, expression of all *FcgRs*, except *FcgRI*, rapidly increased from the baseline as early as 3 h after a single LPS activation (Figure 8A–D). In single LPS-stimulated (N/LPS) WT macrophages, there was a higher expression of *FcgRIIb* with similar *FcgRIII* and *FcgRIV* expression at most time points compared to FcgRIIb-/- cells (Figure 8A–D). Notably, a higher *FcgRIII* expression at 6 h (Figure 8C) and a lower *FcgRIV* expression (Figure 8D) at 3 h of LPS activation in WT macrophages in comparison to FcgRIIb-/- cells were demonstrated.

**Figure 9:**
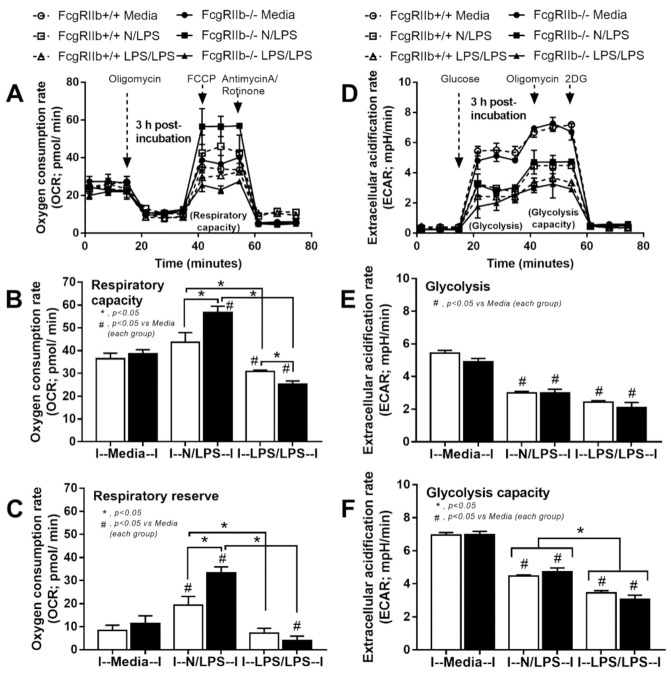
The extracellular flux analysis pattern of macrophages from FcgRIIb-/- lupus mice and wild-type (FcgRIIb+/+) mice after 3 h of the incubation by media control (Media), a single LPS stimulation (N/LPS), and the twice LPS stimulation (LPS/LPS) as demonstrated by mitochondrial stress test (a pattern of oxygen consumption rate, respiratory capacity, and respiratory reserve) (**A–C**) and glycolysis stress test (a pattern of extracellular acidification rate, glycolysis and glycolysis capacity) (**D–F**). Independent triplicate experiments were performed. 2-DG, 2-Deoxy-D-glucose; FCCP, carbonyl cyanide-4-(trifluoromethoxy) phenylhydrazone.

With respect to LPS tolerance (LPS/LPS), *FcgRIIb* and *FcgRIII* in WT macrophages were higher than FcgRIIb-/- cells without the expression of *FcgRI* and *FcgRIV* in both groups (Figure 7E–H). A comparison between single LPS vs. LPS tolerance showed a reduction in *FcgRIIb* and *FcgRIII* in LPS-tolerant WT cells, while only a reduced *FcgRIV* expression in LPS-tolerant FcgRIIb-/- macrophages (Figure 8I-L). In addition, the status of cell energy function was determined, because of the possible impact on cell function [8,47]. Accordingly, a single LPS stimulation enhanced mitochondrial functions with reduced glycolysis activity in macrophages from both mouse strains with a higher level of mitochondria activation in FcgRIIb-/- cells (Figure 9A–F). In LPS tolerance, there was a similar reduction of mitochondria and glycolysis activity (Figure 9A–F), except for the lower respiratory capacity in LPS-tolerant FcgRIIb-/- macrophages compared to the WT cells (Figure 9B).

Nevertheless, the direction of inflammatory responses in N/LPS demonstrated the higher level in FcgRIIb-/- macrophages compared with the WT (Figure 7A,B) in correspondence to the data in mice (Figure 1, Figure 2, Figure 3, Figure 4, Figure 5 and Figure 6). Meanwhile, the inflammatory direction of LPS/LPS macrophages showed the more prominent responses in WT macrophages than the FgRIIb-/- cells (Figure 7C,D) which was opposite to the mouse data (Figure 1, Figure 2, Figure 3, Figure 4, Figure 5 and Figure 6). The discordance between the less inflammatory responses in LPS/LPS FcgRIIb-/- macrophages in vitro and the more prominent inflammation in FcgRIIb-/- mice with NSAIDs-induced endotoxemia implied a possible limited impact of LPS tolerance in NSAIDs-administered mouse model. Unfortunately, the in vivo biomarker for LPS tolerance is still non-established [48,49].

### 2.3. An Anti-Inflammatory Effect of NSAIDs in LPS-Activated Macrophages

**Figure 10:**
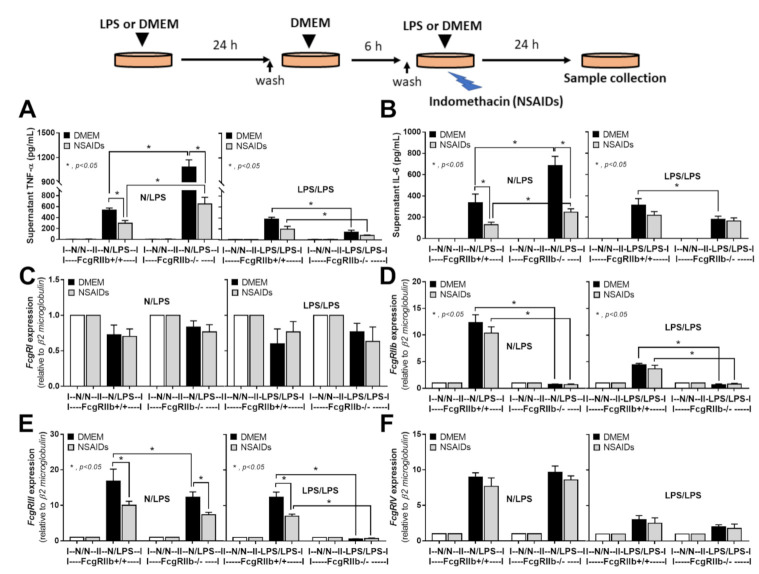
Schema of the in vitro experiments (upper part of figure) and the characteristics of macrophages from FcgRIIb-/- lupus mice and wild-type (FcgRIIb+/+) mice after 6 h incubation with a single lipopolysaccharide stimulation (N/LPS) or stimulation twice (LPS/LPS) with or without indomethacin (NSAIDs) as evaluated by supernatant proinflammatory cytokines (**A**,**B**) and the gene expression of *FcgRs* by quantitative polymerase chain reaction (**C–F**) were demonstrated. Independent triplicate experiments were performed.

As NSAIDs were still continuously administered in mice even after an occurrence of NSAIDs side effect in the mouse model, the impact of NSAIDs on LPS-activated macrophages was possible. Then, indomethacin was incubated in LPS-activated macrophages with both N/LPS and LPS/LPS (Figure 10A–F). With the prominent inflammation in N/LPS, NSAIDs effectively reduced the production of proinflammatory cytokines in both FcgRIIb-/- macrophages and WT cells supporting the NSAIDs anti-inflammatory property [50,51,52] but the level of supernatant cytokines in FcgRIIb-/- macrophages was still higher than the WT (Figure 10A,B). Additionally, NSAIDs in N/LPS activated macrophages reduced only *FcgRIII* expression, but not *FcgRIIb* and *FcgRIV*, (Figure 10C–F) suggesting a possible different influence of cyclooxygenase enzyme or prostaglandins during the gene expression of the different *FcgRs* isoforms [53,54]. On the other hand, in LPS tolerance, NSAIDs did not significantly further reduce cytokines and *FcgRs* in FcgRIIb-/- macrophages while decreased only *FcgRIII* expression in WT macrophages (Figure 10A–F) perhaps due to the low level of the responses.

## 3. Discussion

The elevation of anti-dsDNA in FcgRIIb-/- mice at 24 wks old induced asymptomatic immune complex deposition on intestines that enhanced the susceptibility toward indomethacin-induced enteropathy. Despite the anti-inflammatory properties of NSAIDs, high dosage of indomethacin caused systemic inflammation through the injury induction in several systems, including gut leakage-induced endotoxemia, that possibly exacerbated lupus activity.

### 3.1. Prominent Indomethacin-Induced Nephropathy and Enteropathy in FcgRIIb-/- Mice Compared to Wild-Type Mice: An Impact of Immune Deposition in Asymptomatic Lupus

Due to the necessity of cyclooxygenase (COX) enzyme in the homeostasis of several biological systems, NSAIDs adverse effects are demonstrated in multiorgans especially nephropathy and enteropathy [55]. Among the different manifestations of NSAIDs nephropathy (renal ischemia, cortical necrosis, proteinuria, and interstitial nephritis) [31,32], renal ischemic tubular injury is most common with the subtle histological findings including renal tubular vacuolation and loss of brush borders [56,57,58]. Here, indomethacin in WT mice caused proteinuria with only tubular vacuolization indicating NSAIDs-induced minimal change disease [59] possibly because of podocyte injury from leukotrienes-activated T cells (increased leukotrienes conversion from arachidonic acid due to the blockage of prostaglandin synthesis) [60]. In parallel, NSAIDs administration in 24-wk-old FcgRIIb-/- mice exacerbated lupus nephritis as indicated by red blood cell casts, proteinaceous casts, and glomerular immune complex deposition [36]. Perhaps, NSAIDs induced uremia and uremia-induced inflammation exacerbates lupus activity [34,35]. However, NSAIDs nephropathy might not be a main lupus exacerbating factor in NSAIDs-mouse model because (i) the uremia in NSAIDs model (no obvious renal histological damage) is less severe than other direct renal damage models (ischemia and bilateral nephrectomy) [61,62] and (ii) endotoxemia from uremia (an indirect mucosal damage) is less severe than the endotoxemia from the direct gut mucosal injury caused by NSAIDs [63,64],

On the other hand, NSAIDs induced prominent enteropathy in the upper and lower gastrointestinal tract [65] and caused endotoxemia from the direct gut mucosal injury [66]. More specifically, indomethacin-induced endotoxemia is well-known [67,68], partly due to the enterohepatic drug recycling which results in prolonged and repeated exposure of the compound to the intestinal mucosa. As the gut leakage brought on spontaneous endotoxemia in full-blown FcgRIIb-/- mice at 40 wks old and in patients with active lupus [5], FcgRIIb-/- mice at 24 wks old, which demonstrated only increased anti-dsDNA but not lupus nephritis, were selected for use in the NSAIDs evaluation. Surprisingly, NSAIDs only increased the mortality rate in FcgRIIb-/- lupus mice, but not in the age-matched WT mice, despite the lack of difference in stool characteristics, stool occult blood, and body weights. This suggests that the cause of death in lupus mice was less likely to be massive mucosal bleeding or anorexia, the common NSAIDs adverse effects, but might be, at least in part, due to the more severe systemic inflammation. Accordingly, indomethacin-induced gut leakage was demonstrated. In addition, endotoxin, a potent immune activator, together with systemic cytokines in NSAIDs-administered mice were more predominant in FcgRIIb-/- mice compared to WT mice. NSAIDs induced more severe intestinal mucosal injury in FcgRIIb-/- mice as the ulcers could be demonstrated in all intestinal parts. Meanwhile, only monoclonal cells infiltration occurred in NSAIDs-administered WT mice. The increased susceptibility toward NSAIDs-enteropathy might be due to the intestinal preconditioning injury as demonstrated by the intestinal immune deposition in FcgRIIb-/- mice, but not in WT, at the baseline before NSAIDs administration. The immune deposition in the gut increased with NSAIDs administration in both mouse strains, but was more predominant in FcgRIIb-/- mice. While immune deposition in the gut of NSAIDs-administered WT mice is only for a wound healing process [44], prominent immune deposition in FcgRIIb-/- mice is also due to an increase in lupus antibody production. The increased level of anti-dsDNA in FcgRIIb-/- mice was possibly caused by indomethacin-induced endotoxemia supporting the exacerbation of lupus activity through the active inflammation [34,35]. Furthermore, NSAIDs enteropathy was severe enough to cause local intestinal inflammation in FcgRIIb-/-, but not in WT mice, as indicated by increased cytokines in all intestinal parts.

### 3.2. Prominent Inflammatory Responses against Endotoxin in FcgRIIb-/- Mice over Wild-Type Mice, an Inhibitory Effect of FcgRIIb.

Hyperimmune responsiveness related to a defect in negative signaling is demonstrated in FcgRIIb-/- lupus mice [1] and in macrophages [10]. In addition, LPS is a pathogenic molecule foreign to the host, which potently activates innate immune responses in the host through TLR-4 [69], resulting in systemic inflammatory responses [5,33,70,71,72]. In FcgRIIb-/- macrophages, a profound response (high cytokine production) occurred after the first LPS stimulation followed by an extreme LPS tolerance (low cytokine production) after the second LPS activation when compared to WT cells [8,9,10]. Despite the continuation of endotoxin stimulation, LPS tolerance was not dominant in NSAIDs enteropathy in FcgRIIb-/- mice as indicated by the higher amount of serum cytokines in NSAID-administered FcgRIIb-/- mice compared to WT. However, heterogeneity of macrophages in vivo may exist, so that LPS tolerance might be induced in some cells. We further tested LPS activation in macrophages both with single and double stimulations. Due to the cross-talk between TLR-4 and FcgRs [46] and the balance between activating and inhibitory FcgRs, an alteration of FcgRs might be associated with the direction of the immune response. After a single LPS stimulation, both of the activating FcgRs (*FcgRIII* and *FcgRIV*) and the inhibitory FcgR (*FcgRIIb*) were enhanced in WT macrophages, while only the activating FcgRs (*FcgRIII* and *FcgRIV*), but not the inhibitory *FcgRIIb*, was increased in FcgRIIb-/- cells.

While *FcgRIV* expression in FcgRIIb-/- macrophages was higher than WT cells at 3 h post-LPS stimulation, *FcgRIII* in WT cells was higher than FcgRIIb-/- cells at 6 h of the activation. This suggests a different type of activating FcgRs among WT and lupus cells. It is thought that prominent cytokine production in single LPS-stimulated FcgRIIb-/- macrophages was associated with the enhanced *FcgRIV* without the presence of *FcgRIIb* inhibitory receptors. Mouse FcgRIV is more functionally active than FcgRIII, since FcgRIV recognizes three out of four isoforms of mouse IgG (IgG1, IgG2a, and IgG2b), while FcgRIII recognizes only mouse IgG1 (mouse IgG3 was nonrecognizable by FcgRs) [45,73]. The enhanced activating FcgRs, without an inhibition, might be associated with the higher mitochondrial activity in the single LPS-stimulated FcgRIIb-/- macrophages compared to WT cells. Regarding cell energy, a similar reduction of glycolysis activity in macrophages of both mouse strains occurred after a single LPS stimulation. This result supports that prominent glycolysis utilization during LPS stimulation [74] and the enhanced mitochondrial activity found only in the FcgRIIb-/- macrophages might be responsible for the higher cytokine production. Despite the prominent cytokine production after a single LPS stimulation, LPS tolerance as determined by the difference between a single vs. twice LPS stimulation is more profound in FcgRIIb-/- mice [9,10,47]. Indeed, there was a reduction in expression of activating *FcgRs*, glycolysis capacity, and cytokine production of LPS tolerant FcgRIIb-/- macrophages, when compared to WT cells. Despite the unavailability of LPS tolerant biomarker, serum cytokine of NSAIDs-administered FcgRIIb-/- mice was higher than in WT mice suggesting the non-prominent LPS tolerance in vivo. The lack of LPS tolerance in macrophages in vivo despite a possible continuous LPS activation caused by gut translocation might be due to the macrophage recovery or an insufficient level of endotoxin in the model. Nevertheless, indomethacin demonstrated the anti-inflammatory effect against LPS stimulated macrophages, more prominent in N/LPS when compared with LPS/LPS, as indicated by a reduction of supernatant inflammatory cytokines supporting a role of COX enzyme in proinflammatory macrophages [50]. In parallel, indomethacin reduced only the gene expression of *FcgRIII*, but not other *FcgRs*, which might be associated with the diverse impact of prostaglandin in the synthesis of different FcgRs [54] and the selective blockage of macrophage functions by NSAIDs [75]. However, even with the NSAIDs anti-inflammatory effect, the proinflammatory cytokines and the gene expression of activating *FcgRIII* in FcgRIIb-/- macrophages were also higher than the WT cells implying more prominent inflammatory responses in FcgRIIb-/- mice that might be an exacerbating factor for lupus activity.

Several limitations of the study are noted. First, our study tested only one model of lupus mice focusing on a single gene as the possible cause, when a variety of lupus models from different physiologies exist. Lupus is a considered clinical syndrome with multiple factors and multigene involvement [76]. Second, there is a limitation in the mouse model due to the very high dose of indomethacin compared to a more typical lower dose in patients. Likewise, only indomethacin, a short-acting drug with a high GI side effect, was tested due to its popular utilization in animal models [42,43], despite a variety of newer drugs in the clinical practice. Third, only the gene expression, but not the protein abundance, of *FcgRs* was explored. Fourth, only an association, but not the more physiologic evaluations (cause–effect), between the macrophage metabolic profiles and LPS stimulation was performed. Nevertheless, our data provide a proof of concept that NSAIDs (indomethacin) could induce the inflammatory responses, including gut leakage, that subsequently affects lupus activity. Our initial findings suggest that additional studies in patients are warranted.

## 4. Materials and Methods

### 4.1. Animals and Animal Model

The Institutional Animal Care and Use Committee of the Faculty of Medicine, Chulalongkorn University, Bangkok, Thailand, approved the study (025/ 2562; 1 Oct 2018) under the protocols of the National Institutes of Health (NIH), USA. Only female mice were used in experiments. FcgRIIb deficient mice with a C57BL/6 background (FcgRIIb-/-) were provided by Dr. Silvia Bolland (NIAID, NIH, Maryland, USA). The WT and FcgRIIb-/- mice were separately housed to avoid the effect of allocoprophagy (the consumption of feces from other mice) on the experiments [36,71]. Female wild-type (WT) mice at 8 wks old were purchased from Nomura Siam International (Pathumwan, Bangkok, Thailand) and maintained in the facility until 24 wks old. Since FcgRIIb-/- mice develop anti-dsDNA auto-antibodies as early as 16–24 weeks without kidney injury and have lupus nephritis at 40 wks old [5,6,7,17,18], FcgRIIb-/- mice at 24 wks of age were used as a representative model of asymptomatic lupus. The NSAIDs-induced enteropathy model was induced by daily oral administration of indomethacin (Sigma-Aldrich, St. Louis, MO, USA) at 25 mg/kg diluted in 0.2 mL of phosphate buffer solution (PBS) for 7 days before sample collection. Observation for the 10 days survival analysis followed protocols from previous publications [77,78]. Spot urine collection was performed at 6 h before sacrifice by placing mice in the metabolic cage (Hatteras Instruments, NC, USA). Mice were sacrificed with cardiac puncture under isoflurane anesthesia before the collection of blood, feces (in cecum), and organs. Immunochemical fecal occult blood test was evaluated by ColoScreen (HL-5072) (Helena Thai Laboratories, Bangkok, Thailand). Hematocrit was measured by microhematocrit method with Coulter Counter (Hitachi 917; Boehringer Mannheim, Indianapolis, IN, USA) and total white blood cell (WBC) count was evaluated by a hemocytometer using blood (15 µL) in the hemolysis agent (250 µL of 3% acetic acid) [79]. Liver injury was indicated by serum alanine transaminase (ALT) by EnzyChrom alanine transaminase assay (EALT-100) (Bioassay, Hayward, CA, USA). Assays for serum blood urea nitrogen and creatinine used the QuantiChrom Creatinine-Assay (DICT-500) and QuantiChrom Urea-Assay (DIUR-500) (BioAssay), respectively. Serum cytokines were measured by ELISA (PeproTech, Oldwick, NJ, USA). In addition, serum anti-dsDNA was analyzed following a protocol using coated Calf-DNA (Invitrogen, Carlsbad, CA, USA) [80]. Symptomatic lupus was defined as increased serum anti-dsDNA antibodies with high serum creatinine. Values from the age-matched WT mice served as the control. Mouse organs from both samples were put in 10% formalin or snap frozen and stored separately at −80 °C for the additional analyses. Different intestinal parts, including duodenum (distal to the pyloric sphincter), jejunum (central section of small intestine), ileum (proximal to cecum), and colon (distal to cecum) were collected [81]. The intestines were washed several times in PBS, then weighed, homogenized, and centrifuged for the presence of cytokines in tissue.

### 4.2. Gut Permeability Determination

Fluorescein isothiocyanate-dextran (FITC-dextran), a gut nonabsorbable molecule, was orally administered to determine gut permeability as previously published [70] by orally administered FITC-dextran (molecular weight 4.4 kDa; Sigma-Aldrich) at 25 mg/mL in 0.25 mL PBS at 3 h before sacrifice. Serum FITC-dextran was measured by fluorospectrometry (microplate reader; Thermo Scientific, Wilmington, DE, USA). In addition, serum endotoxin (lipopolysaccharide; LPS) was measured as an additional gut leakage parameter using the HEK-Blue LPS detection (InvivoGen, San Diego, CA, USA). Values of LPS < 0.01 EU/mL were recorded as zero due to the limitation of the standard curve.

### 4.3. Histology Analysis and Immunofluorescent Imaging

The semiquantitative evaluation of renal histology on paraffin-embedded slides was performed with Periodic acid-Schiff (PAS) color at 200× magnification in 10 randomly-selected fields for each animal. Renal injury was defined as tubular epithelial swelling, loss of brush border, vacuolar degeneration, necrotic tubules, cast formation, and desquamation using the following scoring method: 0—area of damage <5%; 1—area of damage 5–10%; 2—area of damage 10–25%; 3—area of damage 25–50%; 4—area of damage >50% [74,82,83].

In parallel, the semiquantitative evaluation of intestinal histology on hematoxylin and eosin (H&E) staining at 200x magnification following a publication [84] was based on mononuclear cell infiltration, epithelial hyperplasia (epithelial cells in longitudinal crypts), reduction of goblet cell and epithelial cell vacuolization. Scoring was assigned using the following thresholds: 0—leukocyte < 5% and no epithelial hyperplasia (<10% of control); 1—leukocyte infiltration 5–10% or hyperplasia 10–25%; 2—leukocyte infiltration 10–25% or hyperplasia 25–50% or reduced goblet cells (>25% of control); 3—leukocyte infiltration 25–50% or hyperplasia >50% or intestinal vacuolization; 4—leukocyte infiltration >50% or ulceration. The immunoglobulin deposition in intestines was visualized by immunofluorescence prepared in Cryogel (Leica Biosystems, Richmond, IL, USA), stained with goat anti-mouse IgG and DAPI (4′,6-diamidino-2-phenylindole), a blue-fluorescent DNA stain (Alexa Fluor 488, Abcam, Cambridge, MA, USA), then detected and analyzed for fluorescent intensity by ZEISS LSM 800 (Carl Zeiss, Germany). While antibody deposition in lupus mice with high anti-dsDNA indicates immune complex deposition [36], antibodies on damaged tissues in indomethacin-induced enteropathy indicate wound repairing processes [44].

### 4.4. Bone Marrow Derived Macrophages and the In Vitro Experiments

Due to (i) the dominant role of macrophages in LPS recognition [5,17,18,36,81], (ii) the more prominent responses against LPS in comparison with self-antigens [15], (iii) the immune hyper-responsiveness of FcgRIIb-/- macrophages [1] and (iv) the differential response of single vs. double LPS stimulation [8,9], both the single and double LPS stimulations were explored in bone marrow-derived macrophages [8,9]. In the double LPS stimulation (LPS/LPS) (a representative LPS tolerance), macrophages (1 × 10^5^ cells/well) were incubated with LPS (*Escherichia coli* 026: B6; Sigma-Aldrich) at 100 ng/mL for 24 h before washed and incubated with DMEM (Dulbecco’s Modified Eagle Medium) for a 6 h neutralization period, then restimulated with 100 ng/mL LPS before supernatant and cell collection. In the single LPS stimulation (N/LPS), macrophages were incubated in DMEM for 24 h, then washed and incubated for 6 h in DMEM, before incubated with LPS. Supernatant cytokines were measured by ELISA (PeproTech) and FcgRs (both activating and inhibitory receptor) in macrophages using real time polymerase chain reaction (PCR). Total RNA and reverse transcription were prepared using an RNA easy mini kit (Qiagen, Hilden, Germany) and a high capacity reverse transcription assay (Applied Biosystems, Warrington, UK), respectively. Real-time PCR was performed using an Applied Biosystems 7500 Real-Time PCR System with SYBR^®^ Green PCR Master Mix (Applied Biosystems) and were demonstrated in terms of relative quantification using the comparative threshold (delta-delta Ct) method (2^−ΔΔCt^) as normalized by β2M (an endogenous housekeeping gene). A list of primers for PCR is shown in Table 1. In addition, energy metabolism profiles, using an estimation of glycolysis and mitochondrial oxidative phosphorylation to determine the extracellular acidification rate (ECAR) and oxygen consumption rate (OCR), respectively, were run with Seahorse XFp Analyzers (Agilent, Santa Clara, CA, USA) on macrophages at 1 × 10^4^ cells/well by Seahorse Wave 2.6 software as previously described [8]. Additionally, indomethacin (Sigma-Aldrich) at 100 µM was added in N/LPS and LPS/LPS to evaluate an in vitro impact of NSAIDs.

### 4.5. Statistical Analysis

Statistical differences among groups were examined using the unpaired Student’s *t*-test or one-way analysis of variance (ANOVA) with Tukey’s comparison test for the analysis of experiments with two groups or more than two groups, respectively. Results are presented with the mean ± standard error (SE). Statistical comparisons of data before and after treatment were conducted by paired Student’s *t*-test. SPSS 11.5 software (SPSS, Chicago, IL, USA) was employed for all statistical analyses.

## 5. Conclusions

**Figure 11:**
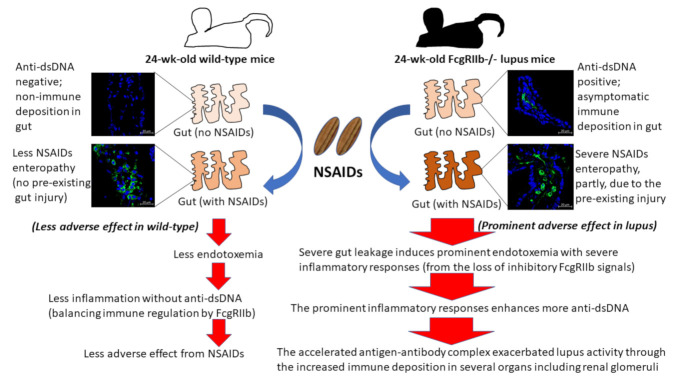
The proposed hypothesis demonstrates a possible main mechanism of NSIADs-exacerbated lupus activity. Asymptomatic immune complex deposition in several organs, especially intestines, enhances a susceptibility to NSAIDs-induced organ injury and the inflammatory responses against the injury stimulates lupus activity. As such, the more severe endotoxemia from prominent gut injury and the endotoxin hyper-responsiveness, partly through the crosstalk between TLR-4 and activating FcgRs [14], exacerbates lupus activities in FcgRIIb-/- mice.

In conclusion, our data support that prominent hyperinflammatory responses in FcgRIIb-/- mice compared to WT mice after NSAIDs administration were due to an inability to produce inhibitory FcgRIIb signaling (Figure 11). Despite the multiple systemic injuries of NSAIDs, the gastrointestinal adverse effect is a most common complication that is resulting in endotoxemia, a potent inflammatory exacerbation factor in lupus [36]. Since (i) gut leakage is demonstrated in patients with lupus [16], (ii) NSAIDs enteropathy enhances gut leakage, and (iii) translocation of pathogens molecules (due to gut leakage) activates lupus [36], the administration of NSAIDs, especially in high doses in patients with lupus, might accelerate the disease activity. Study findings warrant further clinical studies of patients with lupus characterized by gut leakage and high dose NSAIDs.

## Figures and Tables

**Table 1:** List of primers in the study are demonstrated.

Primers	Forward	Reverse
Fc gamma receptor I (*FcgRI*)	5′-CACAAATGCCCTTAGACCAC-3′	5′-ACCCTAGAGTTCCAGGGATG-3′
Fc gamma receptor IIb (*FcgRIIb*)	5′-TTCTCAAGCATCCCGAAGCC-3′	5′-TTCCCAATGCCAAGGGAGAC-3′
Fc gamma receptor III (*FcgRIII*)	5′-AGGGCCTCCATCTGGACTG-3′	5′-GTGGTTCTGGTAATCATGCTCTG-3′
Fc gamma receptor IV (*FcgRIV*)	5′-AACGGCAAAGGCAAGAAGTA-3′	5′-CCGCACAGAGAAATACAGCA-3′
β2 microglobulin (*β2M*)	5′CCACTGAAAAAGATGAGTATGCCT-3′	5′-CCAATCCAAATGCGGCATCTTCA-3′

## Data Availability

The data presented in this study are available on request from the corresponding author.
